# Gerotranscendence Intervention: Insights from a Pilot RCT

**DOI:** 10.1093/geroni/igaf122.3067

**Published:** 2025-12-31

**Authors:** Lia Araujo, Taiane Abreu, Oscar Ribeiro, Laetitia Teixeira

**Affiliations:** RISE-Health, Porto, Porto, Portugal; University of Aveiro, Aveiro, Aveiro, Portugal; University of Aveiro, Aveiro, Aveiro, Portugal; ICBAS-UP, Porto, Porto, Portugal

## Abstract

As the gerotranscendence theory gains visibility in the gerontological field, activities and programs exploring its assumptions with older adults are emerging. Despite the potentialities of the existing interventions, these studies have key limitations, such as inconsistent outcome measurement, lack of randomized controlled trials (RCTs), and insufficient focus on long-term care (LTC) populations. This pilot study aims to present the structure and the benefits of a gerotranscendence program developed with older adults in LTC settings. Forty-four older adults (23 in the experimental group and 21 in the control) have participated in a RCT of a gerotranscendence intervention program, with six weekly sessions, conducted in four different LTC facilities. A protocol with the Gerotranscendence Scale (GST2), Satisfaction with Life Scale (SWLS) and the Geriatric Depression Scale (GDS-15) was used at the beginning and at the end of the intervention. After the post-test, two focus groups were conducted with participants from the experimental group to gather their views on the intervention and how gerotranscendence theory applied to their experiences. Although quantitative analyses revealed no significant changes in the outcome measures (GDS, SWLS, and GST2), qualitative findings provided valuable insights into participants’ perceptions and experiences with gerotranscendence, highlighting their redefined views on aging, the benefits of self-reflection, the importance of group dynamics, and the challenges of aging and living in LTC facilities. This study addresses a critical gap in the literature by exploring the structure and impact of a gerotranscendence program designed for older adults in LTC settings, using a mixed-methods approach.

